# IL-1α is required for T cell-driven weight loss after respiratory viral infection

**DOI:** 10.1016/j.mucimm.2024.02.005

**Published:** 2024-04

**Authors:** Ziyin Wang, Leah F. Cuthbertson, Chubicka Thomas, Hadijatou J Sallah, Lucy G. Mosscrop, Haoyuan Li, Tiina Talts, Kartik Kumar, Miriam F. Moffatt, John S. Tregoning

**Affiliations:** 1 Department of Infectious Disease, St. Mary's Campus, Imperial College London, UK; 2 National Heart and Lung Institute, Imperial College London, UK; 3 Virus Reference Department, Public Health Microbiology, United Kingdom Health Security Agency, London, UK

## Abstract

Respiratory viral infections remain a major cause of hospitalization and death worldwide. Patients with respiratory infections often lose weight. While acute weight loss is speculated to be a tolerance mechanism to limit pathogen growth, severe weight loss following infection can cause quality of life deterioration. Despite the clinical relevance of respiratory infection-induced weight loss, its mechanism is not yet completely understood. We utilized a model of CD 8^+^ T cell-driven weight loss during respiratory syncytial virus (RSV) infection to dissect the immune regulation of post-infection weight loss. Supporting previous data, bulk RNA sequencing indicated significant enrichment of the interleukin (IL)-1 signaling pathway after RSV infection. Despite increased viral load, infection-associated weight loss was significantly reduced after IL-1α (but not IL-1β) blockade. IL-1α depletion resulted in a reversal of the gut microbiota changes observed following RSV infection. Direct nasal instillation of IL-1α also caused weight loss. Of note, we detected IL-1α in the brain after either infection or nasal delivery. This was associated with changes in genes controlling appetite after RSV infection and corresponding changes in signaling molecules such as leptin and growth/differentiation factor 15. Together, these findings indicate a lung-brain-gut signaling axis for IL-1α in regulating weight loss after RSV infection.

## Graphical abstract

**Figure:**
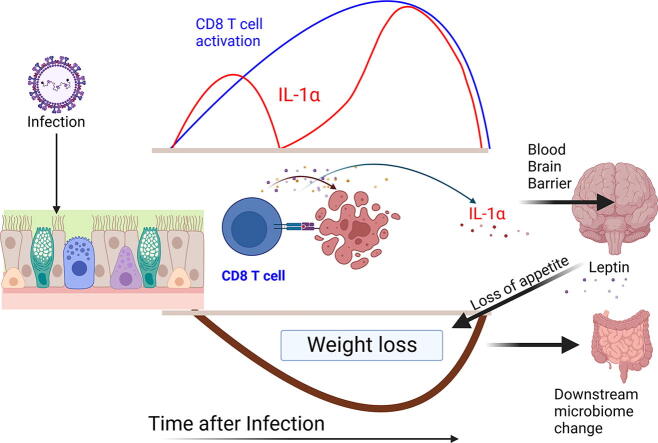

## INTRODUCTION

Respiratory syncytial virus (RSV) is the leading cause of severe lower respiratory illness under the age of 5 years. It affects 33.1 million children per year worldwide, with 3.2 million hospital admissions and 59,600 in-hospital deaths^1^. It also causes 177,000 cases of the elderly every year in the US alone^2^. One of the major challenges in RSV is the weight loss that manifests alongside infection^3^. While the rollout of new vaccines and antibodies is extremely promising, there will continue to be infections and deaths, particularly in low-income countries. In infants with RSV bronchiolitis, poor feeding, and subsequent weight loss during hospitalization are major risk factors for pediatric intensive care unit admission including longer stay lengths^4^. In adults over 65 years, severe weight loss during RSV infection aggravates the underlying condition and is associated with further complications such as congestive heart failure^3^. Clinically, the systematic reduction of host body weight and muscle mass (neither being reversible by conventional nutritional support) is defined as cachexia. Cachexia has also been observed in other respiratory infections including but not limited to influenza^5^ and SARS-CoV-2^6^. Treatment options that have been explored have focused on agonists of appetite-reducing hormones such as ghrelin, but specific anti-cachectic therapies to improve weight loss for respiratory infections do not exist^7^. Consequently, there is a high medical need to alleviate the long-term effects of infection-associated cachexia.

Little is known about the mechanism of respiratory viral infection-associated cachexia. The majority of our understanding of cachexia is derived from cancer studies and lipopolysaccharide (LPS) models^8^, ^9^. Such studies indicate that immune-associated weight loss is driven by the release of inflammatory cytokines; high levels of tumor necrosis factor (TNF), IL-1, IL-6, and interferon-g (IFN-g) are found in the serum of cancer patients^10^. Pro-inflammatory cytokines activate the JAK-STAT and NF-kB signaling pathways, which then play a role in catabolism in adipocytes and muscle cells^10^. TNF activates adipose tissue wasting through the inhibition of peripheral lipoprotein lipase and stimulates lipolysis in adipocytes^11^. Cytokines have also been shown to promote weight loss by penetrating the blood-brain barrier (BBB) and entering the central nervous system (CNS)^12^. In mouse models of human RSV (hRSV) infection, viral replication in the nasal passages and lungs^13^ activates the first line of defense and recruits an initial influx of natural killer (NK) cells, neutrophils, and macrophages to the site of infection. An elevated level of pro-inflammatory mediators such as IL-10 and IFN-g is observed, followed by recruitment of helper CD4^+^ and cytotoxic CD8^+^ lymphocytes. Animals exhibit peak weight loss at day 7 downstream of the lymphocyte-associated inflammation. RSV infection in mice can induce airway obstruction and airway hyper-responsiveness in response to methacholine^13^. In animal models and healthy volunteers challenged with RSV, IL-6, IL-1, and TNF are rapidly detected in bronchoalveolar lavage (BAL) fluid, nasal wash, and serum^13^, ^14^. IL-1 levels are elevated in the nasal mucosa^15^ and the lungs of RSV-infected children^16^. Of note, increased nasal concentrations of IL-1a are predominantly associated with the need for ventilation in children with RSV LRTI^17^. Whether these cytokines affect appetite during RSV infection has not been studied in detail.

As well as understanding the function of pro-inflammatory cytokines during infection-associated cachexia, another key question is the mechanism by which these cytokines are released. Depleting CD8^+^ T cells has been shown to reverse RSV cachexia^18^ and they have been shown to play a role in a chronic lymphocytic choriomeningitis virus infection model^10^. Mounting evidence indicates an important role of CD8^+^ T cells in infants and young children following a severe RSV infection. While both CD4^+^ and CD8^+^ T cells contribute to RSV-induced disease following primary infection, CD8^+^ T cells appear to play the dominant role in viral clearance and weight loss^19^. The frequency of CD8^+^ T cells is increased in tracheal aspirates^20^ and nasal aspirates^21^. RSV-specific CD8^+^ T cells have elevated expression levels of activation markers CD38 and HLA-DR, the proliferation marker Ki-67, and granzyme B^21^. Collectively these studies suggest cachexia-associated cytokines act in consort with immune cells and that CD8^+^ T cells are key in driving cachexia after viral infection. Although CD8^+^ T cells are well-established as key players in mediating viral clearance following acute infection in the lung, a significant gap in our knowledge is how they drive weight loss in the context of respiratory viral infection.

In the present study, we have used an RSV model to identify the downstream factors that cause weight loss following respiratory viral infection. Through RNAseq analysis, we reveal an important role in the IL-1 pathway and go on to show that IL-1α, not IL-1β, plays a key role in CD8-mediated weight loss following RSV infection. Expression data highlights links to leptin and growth/differentiation factor 15 (GDF15) as important downstream factors associated with weight loss. Our data indicates that following RSV infection, IL-1α plays an important role in the lung-gut-brain axis leading to induction of weight loss.

## RESULTS

### Weight loss following RSV infection is driven by CD8 T cells

**Fig. 1:**
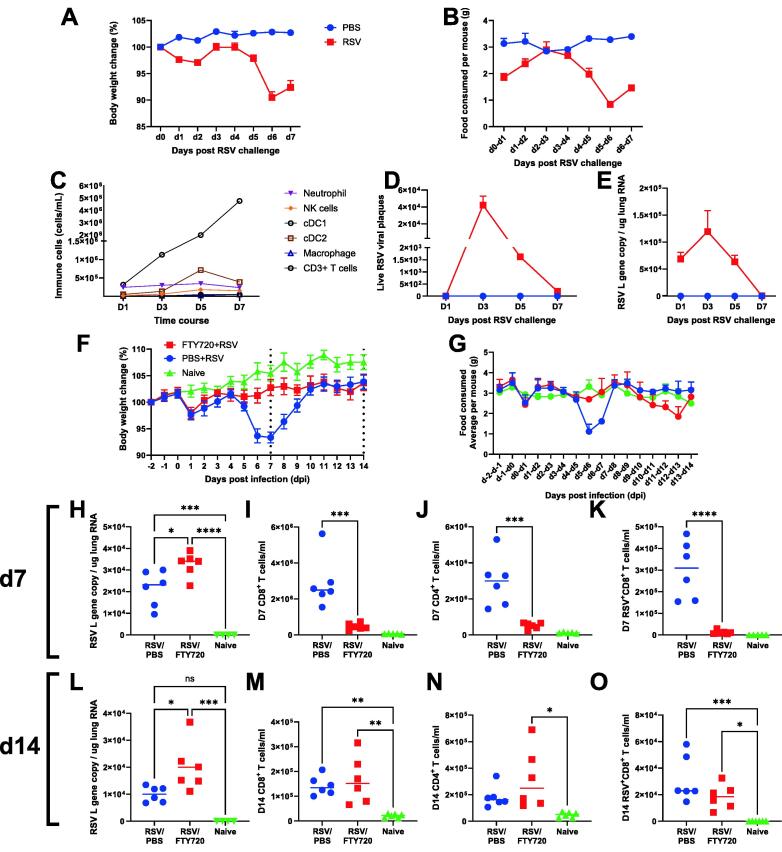
Timing of T cell recruitment to lungs affects disease timing in RSV. 6-7 weeks old BALB/c SPF mice were intranasally infected 7.7 x 10^6^ PFU/ml RSV A2 subtype and culled on day 1, 3, 5, and 7 post infection. Body weight (A) and food consumed (B) were monitored daily. Immune cell populations were measured using flow cytometry (C) at each time course, and live viral plaques (D) and RSV L gene (E) were quantified. 6-7 weeks old BALB/c SPF mice were injected with 25µg of FTY720 or PBS only daily from day -2 to day 6. Mice were intranasally infected with 7.7 x 10^6^ PFU/ml RSV infection on day 0. Body weight (F) and food (G) were monitored daily from day -2 to day 14. At day 7, viral load was determined using RSV L gene qPCR on RNA extracted from the left lung lobe (H) and flow cytometry was used to analyze the number of CD8 T cell (I), CD4 T cells (J) and antigen-specific CD8^+^ T cells (K). At day 14, viral load was determined using RSV L gene qPCR (L) and flow cytometry was used to analyze the number of CD8 T cell (M), CD4 T cells (N) and antigen-specific CD8 T cells (O). N = 5, each dot represents an individual mouse (H-O); or mean +/-SEM –A - E, F-G). Significance calculated by ordinary one-way ANOVA and post test. *P  ≤  0.05, **P  ≤  0.01, ***P  ≤  0.001. Experiment was repeated twice.

It is well established that there is an immune component to weight loss following RSV infection in mice. We first performed a time-course experiment to profile the immune components in the lung as weight loss progressed following RSV infection. The 6 to 8 weeks old BALB/c mice were infected with RSV; body weight (Fig. 1A) and food consumption (Fig. 1B) were monitored daily, and mice were culled at days 1, 3, 5, and 7 post-infection. Innate immune cells were recruited to the site of infection during the early stage of infection with the peak of innate cells coinciding with viral load but returning to basal level on day 7 (Fig. 1C). As innate cell populations decreased, CD3^+^ T cell levels increased sharply from day 5. Weight loss, alongside inappetence, arose simultaneously with T cell response independent of viral burden on day 7, measured by both infectious plaque assay (Fig. 1D) and quantitative polymerase chain reaction (qPCR) (Fig. 1E), consistent with published studies^22^, ^23^. Viral load by the two different assays correlated tightly (R = 0.8).

One remaining question is whether the kinetics of CD8 recruitment to the lung affect the kinetics of weight loss. To explore this, we used FTY720, a sphingosine-1 phosphate receptor modulator that blocks T cell egress from lymphoid tissues and thereby sequesters recirculating T cells within secondary lymphoid organs^17^. The 6–8 weeks old BALB/c SPF mice were intraperitoneally given FTY720 (25 mg) daily from day -2 to day 6 of RSV infection to prevent the recruitment of CD8^+^ T cells from the lymph node to the lungs. The FTY720 treated group did not lose weight at day 7 after infection but did lose weight on day 13. This was reflected by the food consumed data (Fig. 1G), FTY720-treated mice ate less on days 12 to 13 (an average of 1.9 g consumed by FTY720 mice compared to 3g in controls). Mice were culled at days 7 and 14 to examine the viral load and immune response by qPCR and flow cytometry, respectively. At day 7, RSV viral load was higher in FTY720 treated mice compared to control mice (Fig. 1H) and there were significantly fewer CD8^+^, CD4^+^, and RSV-specific CD8^+^ T cells in the lung (Figs. 1I–1K). At day 14, no differences were seen in the number of CD8^+^, CD4^+^ cells, or RSV-specific CD8^+^ T cells in the lungs of mice with and without FTY720 treatment despite a difference in viral load between the two groups (Figs. 1L–1O). These data indicate that the timing of CD8^+^ T cell recruitment into the lungs is associated with weight loss after RSV infection. We therefore set out to understand how CD8^+^ T cells drive weight loss.

### Blood transcriptomics reveals a key role of IL-1 in the CD8 T cell-driven post-viral weight loss

We^18^, and others^20^, ^23^, have previously observed that CD8 T cell depletion reduces weight loss following RSV infection. To explore the mechanism further, transcriptomic changes in the blood after CD8 depletion during RSV infection were investigated. We first re-confirmed that CD8 depletion reduced weight loss. Anti-CD8α monoclonal antibody was intraperitoneally injected on days −1, 2, and 5 of RSV infection. Control mice were injected at the same times with an immunoglobulin (Ig)G isotype control antibody. No weight loss was observed in the naïve group while RSV-infected control mice lost significant weight on days 6 and 7 after infection compared to naïve (Supplementary Fig. 2A). This was reflected in decreased food consumption (average 0.6 g per RSV-infected control mouse on day 6–7 [Supplementary Fig. 2B] compared to 3g on D0-D1). Interestingly, no difference was observed in water intake per mouse for any group (Supplementary Fig. 2C). CD8^+^ T cell depletion was confirmed using flow cytometry in the lung and bronchoalveolar fluid (BAL) of the CD8^+^ T cell-depleted mice (Supplementary Fig. 2D). RSV viral load in the αCD8α group was significantly higher than the control group (Supplementary Fig. 2E) with no virus detected in the naïve group (data not shown).

**Fig. 2:**
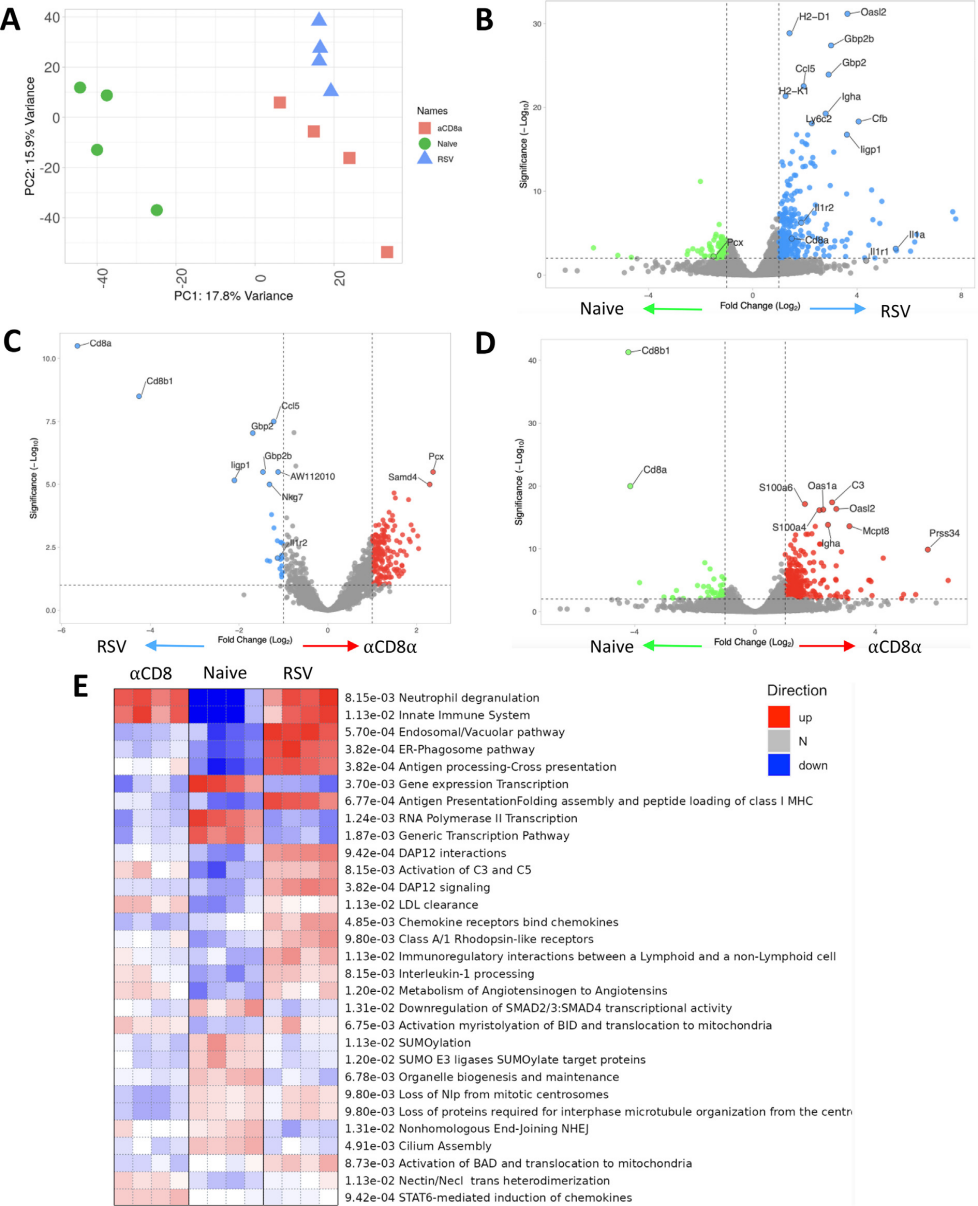
CD8 depletion significantly alters the blood transcriptome following RSV infection. Blood RNA isolated from RSV infected mice with and without CD8 α monoclonal antibody (and naïve control) was analysed by RNASeq. Principle component analysis was carried out to identify batch effects (A). Differential gene expression in mice infected with RSV compared with naïve (B), α CD8 treated mice compared to RSV infection control (C), and α CD8 monoclonal treated mice compared to naïve (D). PGSEA analysis of the groups using Reactome curated data (E). Data from N=4 mice in each group.

Having confirmed our previous findings that CD8 depletion reduces RSV-associated weight loss, blood RNA transcriptomic analysis was used to assess the mechanistic effect that CD8^+^ T cells have on weight loss. Total RNA was extracted from blood samples collected at the peak of T cell response (day 7). Expression data were available for 24,029 genes and principal component analysis (PCA) of the combined data were carried out (Fig. 2A). The PCA analysis revealed that the transcriptomes of αCD8α treated RSV-infected mice (hereafter referred to as αCD8α) form a separate cluster to RSV infection alone (thereby referred to as RSV), with both clustering away from naïve mice indicating the impact of infection on the transcriptome.

Comparing RSV to naïve (Fig. 2B), differential gene analysis revealed 952 differentially expressed genes (DEGs; adjusted *p* value < 0.05, abs(log) fold change > 0.5; Supplementary Table 1). Out of these DEGs, upregulated genes included Guanylate binding proteins (*Gbp2*, *Gbp2b*, *Gbp3*), neutrophil-associated genes (*Ly6a*, *Ly6G*, *Ly6I*, *Ly6c2*), adaptive immunity (*CD8α*, *H2-K1*) and cytokines/chemokine (*Ccl5, Il15*, *Il6, IL-1α*) consistent with previous publications^24^.

Comparing the αCD8α and RSV groups (Fig. 2C) there were 376 DEGs (T Supplementary Table 2). These included CD8 T cell-related genes (*CD8α*, H2-D1, H2-Q6, *Ccl5*, *Ligp1, NKG7,* and *GzmB*) and genes involved in the antiviral response (*Stat1*, *Ifit3*, *Ifitm1*, *Ifi47*, *Oasl2*, *Xcr1*) with both sets of genes exhibiting significantly lower levels of expression in the αCD8α group. Interestingly there were genes upregulated in the αCD8α compared to RSV, including ones associated with Pyruvate metabolism (*Pcx*), zinc finger proteins (*Zfp62, Zfp874a, Zfp773*), and signaling transduction (*Stat5a*). We also compared the αCD8α group to the naïve group; upregulated genes involved in antiviral response were identified including the S100 family (*S100a6, S100a4*) and IFN stimulated genes (*Oas1a* and *Oasl2*) (Fig. 2D); indicating that there was still an antiviral response in the absence of CD8 cells.

We hypothesized that genes upregulated in the αCD8α and RSV groups compared to naïve are responding to infection, but not necessarily driving weight loss (Fig. 2E), while genes that are upregulated in RSV-infected but down in CD8-depleted mice were associated with infection-driven weight loss. Taking this approach, 48 genes were upregulated in both the CD8-depleted and RSV-infected groups, which we would assign to the response to infection (Supplementary Table 3). To identify functional patterns of gene expression, we performed parametric gene set enrichment analysis (PGSEA) using the Reactome database. Overlapping pathways between the αCD8α and RSV groups included activation of innate immune system and neutrophil degranulation (Fig. 2E). This is supported by studies that show neutrophil depletion during RSV infection has no impact on weight loss^25^.

There were 126 genes in the weight loss gene signature (up in αCD8α, down in RSV), this included *Adipor1, Pcx,* and *Slc16a6*. Among the Reactome pathways were “Chemokine receptors bind chemokines” and “Interleukin-1 processing” which included *Rela, Nfkb2, Casp1, Il1b, Nfkb1, Ctsg* (Fig. 2E). The results from this transcriptomics analysis indicate that there is a core response to infection, but only specific aspects are associated with CD8-driven weight loss most notably the inflammatory response.

### IL-1α contributes to RSV-associated weight loss by expanding CD8 T cells and downregulating metabolic pathways

**Fig. 3:**
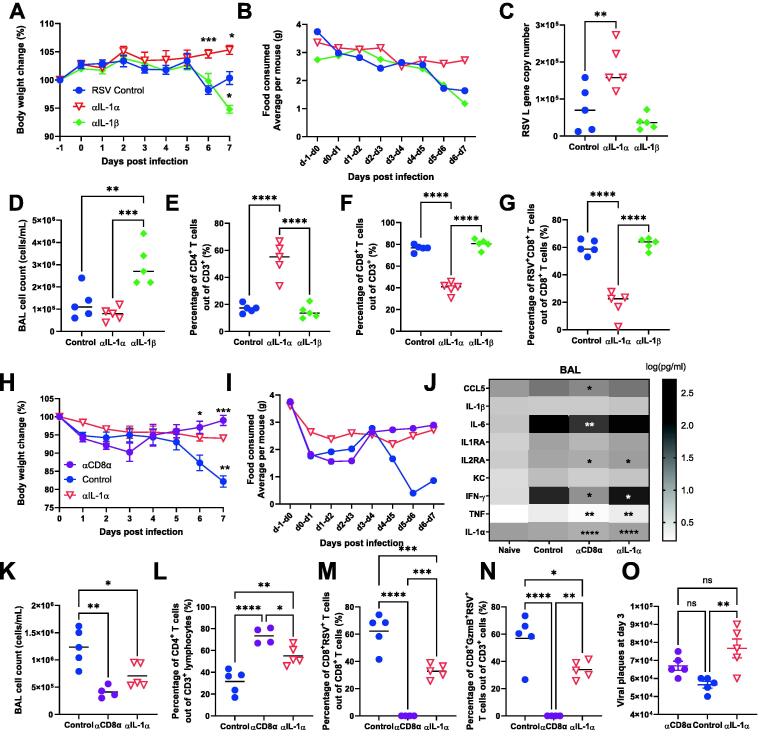
IL-1 α but not IL-1 β blockade reduces weight loss following RSV infection. 6-7 weeks old BALB/c SPF mice were injected intraperitoneally with antibody neutralizing IL-1 α , IL-1β or control IgG on day -1, 1, 3 and 5 post infection. Body weight (A) and food (B) were monitored daily throughout infection. (C) Viral load was determined using RNA extracted from the left lung lobe. Cell count from bronchoalveolar lavage (D) and flow cytometry was performed to determine the percentage of CD4^+^ T cells (E), CD8^+^ T cells (F) and CD8^+^ RSV^+^ T cells (G). 6-7 weeks old BALB/c SPF mice were injected intraperitoneally with antibody depleting CD8 α or neutralizing IL-1 α , or control IgG on day -1, 2 and 5; or -1, 3 and 5 post infection. Body weight (H) and food (I) were monitored daily throughout infection. BAL supernatant was analysed for cytokine by multiplex bead assay (J). Cell counts from BAL (K) and flow cytometry was performed to determine the percentage of CD4^+^ T cells (L), CD8^+^ T cells (M) and CD8^+^ RSV^+^ T cells (N). Viral load measured using viral plaques were performed on day 3 post infection (O). N≥4 per group each dot represents an individual mouse (C-G, K-0); or mean +/-SEM (A, B, H-I). Significance calculated by ordinary one-way ANOVA and post test. *P  ≤  0.05, **P  ≤  0.01, ***P  ≤  0.001, ****P  ≤  0.0001. Experiment was repeated three times.

As one of the pathways highlighted by the PGSEA enrichment analysis was the “Interleukin-1 processing” pathway, and we saw significantly more IL-1 in the lung in previous studies^18^, we next explored the role of the most prominent members of the IL-1 superfamily, IL-1α, and IL-1β, in relation to weight loss after viral infection. Mice were injected intraperitoneally with anti-IL-1α, anti-IL-1β, or control antibody on days −1, 1, 3, and 5 of infection. RSV-infected isotype control mice lost 5% to 10% body weight (Fig. 3A) and their average food intake was reduced on day 6 after infection (Fig. 3B). In contrast, blockade of IL-1α (aIL-1a mice) significantly reduced weight loss with no change in food intake after infection. Mice treated with anti-IL-1β antibody lost more weight on day 7 compared to control or anti-IL-1α treated mice. Anti-IL-1β treated mice had reduced food intake on days 6 to 7 (an average of 1.2 g per mouse compared to 1.6 g per mouse in the control group). This suggests that IL-1β has a protective role during the later stage of infection and is possibly why RSV has evolved proteins to suppress it^26^. Similar to that observed in αCD8α treated mice (Supplementary Fig. 2E), anti-IL-1α mice had significantly more RSV viral load compared to control (Fig. 3C), suggesting that IL-1α could be involved in viral clearance. Flow cytometry was used to examine the T cell populations in the airways (measured in the BAL). Reflecting the increased weight loss, BAL cell count counts were significantly higher in the anti- IL-1β group (aIL-1b) compared to control and aIL-1a mice groups (Fig. 3D). There was a significant increase in the proportion of CD4^+^ T cells (Fig. 3E) and a reduction in the percentage of CD8^+^ T cells (Fig. 3F) in the aIL-1a group. There was also a lower percentage of RSV-specific CD8^+^ T cells (Fig. 3G) recruited to the airways of the anti-IL-1α group compared to control.

As IL-1α neutralization rescued weight loss, we next compared it directly with CD8α depletion. Mice were treated with αCD8α, aIL-1a, or IgG monoclonal antibody as control. Neither the CD8α nor the IL-1α treated mice lost weight (Fig. 3H) or had changes in food consumption following infection (Fig. 3I). In the anti-CD8 treated group, compared to RSV infection there was a significant reduction in airway levels of several cytokines including CCL5, IL-6, IL2RA, IFN-γ, TNF, and IL-1α; in the anti-IL-1α group, there was also a reduction in IL2RA, IFNγ, TNF and IL-1α (Fig. 3J). This was associated with a reduction of cell recruitment, both anti-CD8 and anti-IL-1α treated animals had fewer cells in BAL samples (Fig. 3K). The two groups also had an increased proportion of CD4^+^ T cells (Fig. 3L). A clear reduction in the proportion of RSV-specific CD8^+^T cells was observed in the anti-IL-1α treated group compared to the control group in the BAL (Fig. 3M). The serine protease granzyme B (GzmB) was included as a marker for the cytotoxic activation of the CD8-mediated cell death pathway^27^. There was a notable and significant reduction in GzmB^+^RSV^+^CD8^+^ T cells suggesting that IL-1α acts on the recruitment of antigen-specific CD8^+^ T cells during infection (Fig. 3N). Lastly, we also compared the effect of CD8 T cell depletion or IL-1α blockade on viral load at day 3, when viral load was shown to peak in the lung. We found that IL-1α was contributing to viral clearance very early on, perhaps through innate immune cells (Fig. 3O).

**Fig. 4:**
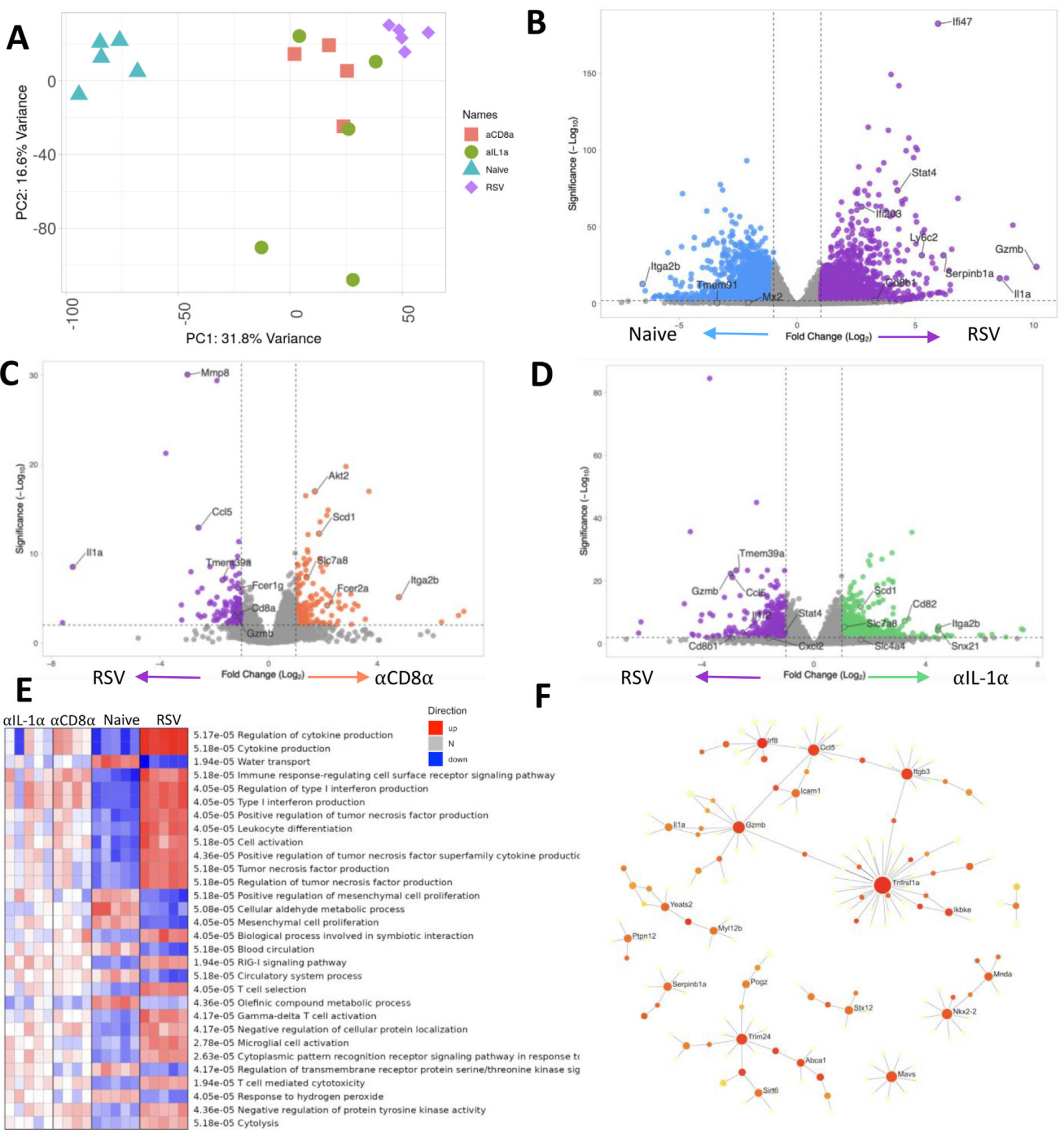
IL-1 α and CD8 depletion induce similar changes in lung transcriptomic signatures following RSV infection. Lung RNA extracted from mice receiving α CD8 α , α IL-1 α or IgG monoclonal antibody infected with RSV (RSV) as well as naïve (no treatment) underwent bulk RNASeq. Principal component analysis was carried out to identify any batch effect using the package pca and function prcomp in R (A). Volcano plot showing genes differentially expressed in mice infected with RSV compared with naïve (B), α CD8 α monoclonal treated mice compared to RSV infected control (C), α IL-1 α monoclonal antibody treated mice compared to RSV infected control (D) All plots generated using package Enhancedvolcano packagIn R). (E) PGSEA analysis of the groups using Reactome curated dataset in R version 1.30.0. (F) 102 significant differentially expressed genes (DEGs) important for inducing weight loss response were identified as up regulated in RSV compared to naïve and downregulated in both α CD8 α treated and α IL-1 α treated compared to RSV. The significant DEGs were used for Network analysis using NetworkAnalyst (version 3.0) using InnateDB curated interactions. Red colors indicate an increasing number of connections.

Having seen a similar effect of both αCD8α depletion and aIL-1a blockade on weight loss following RSV infection, we investigated whether there were similar changes in gene expression following RSV infection in the context of depletion. As it is the site of infection for these studies we looked at transcriptional changes in the lung. Expression data from lung tissue was available for 23,844 genes. The RSV and naïve groups formed separate clusters on PCA and the transcriptomes of αCD8α treated RSV-infected mice formed a cluster with those of the IL-1α treated mice, suggesting that the two treatments resulted in a similar lung transcription profile (Fig. 4A). There were 8999 differential expressed genes in RSV-infected mouse lungs compared to naïve (Supplementary Table 4), many of the DEG were consistent with prior published literature^28^, ^29^ (Fig. 4B). Upregulated genes were associated with the host immune response, for example, *Ifi27*, *Ifi203*, *Gbp1*, *Ifi44l*, *Ifi44*, *Mx1*, *Ly6c2,* and *Ifi47;* these are also present in human *in vivo* studies challenged with RSV^24^. Following CD8 depletion (Fig. 4C) or IL-1α blockade (Fig. 4D), a shift in dynamic metabolic programming was seen after RSV infection. Genes involved in sphingolipid signaling (*Fcer1g*, *Gnai3*), platelet activation (*Itga2b*, *Fgb*, *Kng1*), adipocytokine signaling pathway (*Akt2*, *Jak2*) and metabolic pathways (*Scd1*, *Slc7a8)* were all upregulated (Supplementary Table 5 for CD8α, Supplementary Table 6 for IL-1α). Compared to RSV infection, anti-CD8 and anti-IL-1α treatment reduced the expression of genes in several Reactome pathways including cytokine production, type I IFN production, and TNF production (Fig. 4E), indicating that inflammation plays a key role in post-RSV weight loss. We performed network analysis using InnateDB as an alternative approach to group the 272 DEGs that were associated with weight loss (up in RSV, but down in aIL-1a and αCD8α). We identified several hubs of key genes*, Serpinb1a*, *Tnfrsf1a*, *Trim24*, *Mavs,* and *GzmB* (Fig. 4F) (Supplementary Table 9).

### IL-1α alone induces weight loss via mediating similar immune-related signatures to RSV infection in the lung

**Fig. 5:**
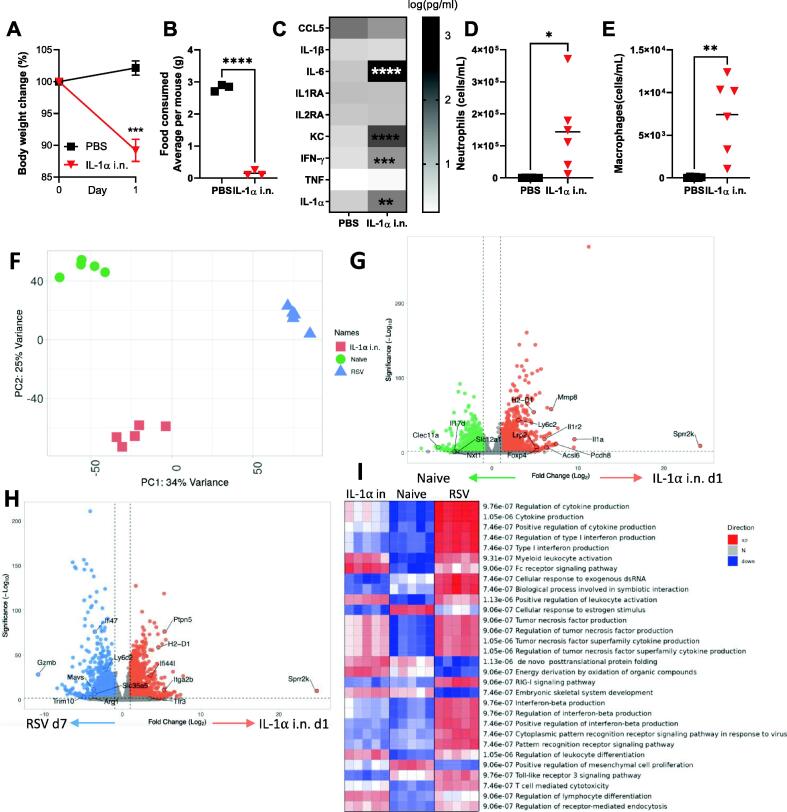
Intranasal administration of IL-1 α induces weight loss and induces similar global signatures in the lung as RSV infection.6-7 weeks old BALB/c SPF mice had 3 mg of recombinant IL-1 α protein or PBS introduced intranasally on day 0. Body weight (A) and food (B) were monitored. Lung supernatant analysed by multiplex cytokine bead assay. Cells were collected from bronchoalveolar lavage (BAL) (C) and flow cytometry was performed to determine the number of neutrophils (D) and macrophages (E). RNASeq was performed on lung RNA extracted from mice day 7 after intranasal administration, RSV or control. Principal component analysis of three conditions (F). Differentially expressed genes in mice administered with IL-1 α compared with naïve (G), IL-1 α administered mice compared to RSV infection control (H). PGSEA analysis of the groups using Reactome curated dataset (I). N≥5, each dot represents an individual mouse (D, E, F, I); or mean +/-SEM (A, G, H); or mean of 2 co-housed mice (B). Significance calculated by ordinary one-way ANOVA and post test. *P  ≤  0.05, **P  ≤  0.01, ***P  ≤  0.001, ****P  ≤  0.0001. Experiment was repeated twice.

Having seen that IL-1α was necessary for weight loss following infection, we wanted to determine whether IL-1α in the lungs was sufficient to cause weight loss. Mice (n = 6) received 3mg of recombinant IL-1α protein intranasally (i.n) on day 0 and their body weight and food intake were monitored; mice were housed in three pairs to enable analysis of food intake. Control mice received phosphate buffered solution (PBS) i.n Mice receiving recombinant IL-1α protein lost 15% of their body weight 24 hours after intranasal challenge (Fig. 5A) and stopped eating on day 1 after treatment (Fig. 5B). As well as significantly elevating IL-1α, intranasal IL-1α administration led to a significantly higher level of a range of other pro-inflammatory cytokines including IL-6, IFN-g, and KC in the lung (Fig. 5C). An influx of immune cells was observed in the BAL, including neutrophils (Fig. 5D) and macrophages (Fig. 5E). Consistent with its established role as a major “alarmin” upstream of pro-inflammatory cytokine and chemokines, intranasal IL-1α recruited innate immune cells and led weight loss through appetite supression^30^.

We next compared the transcriptomic profile in the lungs induced by IL-1α alone to RSV infection. RNA was extracted from the lungs of IL-1α i.n treated animals 1 day after administration and compared to d7 of RSV infection (the peak of weight loss). Expression data were available for 24,345 genes. The transcriptomes of IL-1α treated mice formed a distinct separate cluster to the RSV infection group by PCA (Fig. 5F). Key genes driving the PCA separation include *Trim40*, *Mmp8,* and *GzmB*. Differential gene analysis was performed comparing IL-1α i.n to naïve; a clear upregulation of IL-1-associated genes was observed (Fig. 5G). While there were some differences between IL-1α and RSV (Fig. 5G), out of 3074 genes upregulated in IL-1α i.n compared to naïve 1377 of them were also upregulated in RSV compared to naïve (Supplementary Table 8). This suggested a large overlap in transcriptional change in the lung after infection or IL-1α instillation. Genes upregulated in both IL-1α i.n and RSV infection compared to naïve were involved in IL-1 signaling (*Traf6, IL1r2, IL1b, Il1a, Myd88*) and T cell mediated cytotoxicity (*Akt1, Cd4, Rela, Zap70, Pik3r3, Mapk3, Cdc42, CD8a, Ftn, Ptprc*). However, differences were observed between IL-1α i.n and RSV infection in pathways such as Interferon-beta production, cytoplasmic pattern recognition receptor signaling pathway in response to virus, and pattern recognition receptor signaling pathway (Fig. 5I). These differences reflect the antiviral response to RSV and suggest that these paths do not directly contribute to weight loss following infection.

### Depletion of IL-1α reverses changes in the gut microbiome induced by RSV infection

**Fig. 6:**
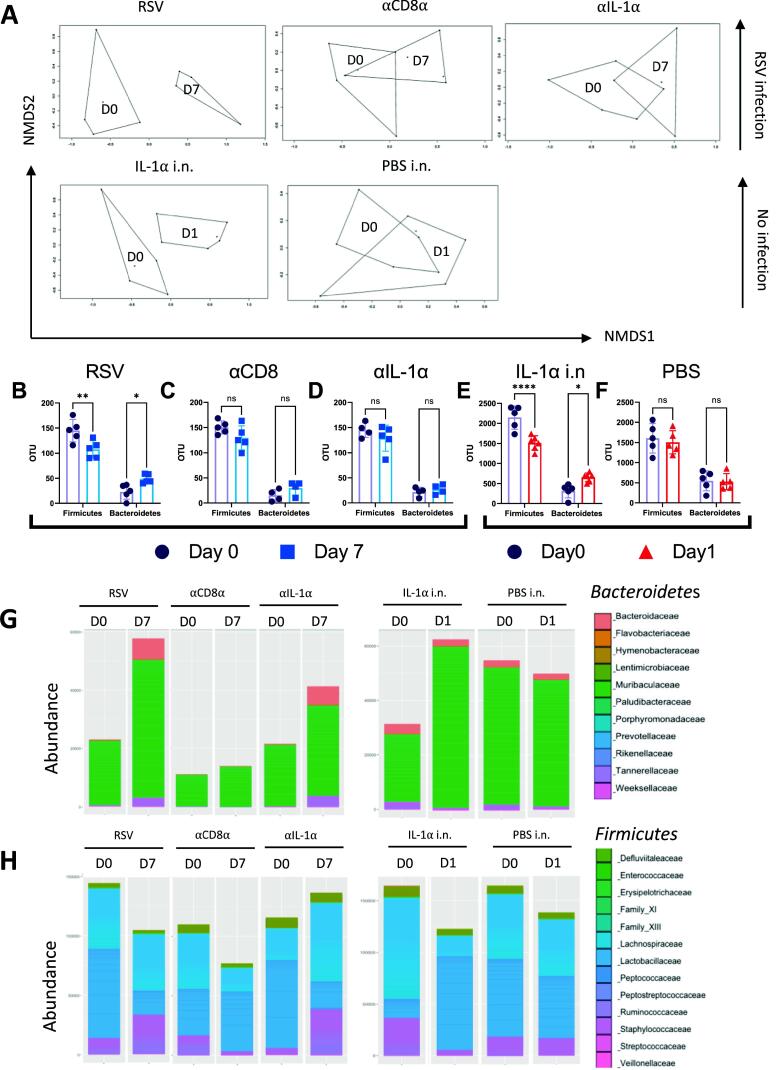
Depletion of IL-1 α reverses the changes in the gut microbiome induced by RSV infection. Change in beta diversity visualized using NMDS on Brays-Curtis dissimilarity matrix for mice received RSV, α CD8 α , α IL-1 α antibody treatment, as well as IL-1 α intranasally (i.n.) administered or PBS (A). ASV abundance of *Bacteroidetes* or *Firmicutes* at phyla level for RSV (B), α CD8 α treated (C), α IL-1 α treated (I IL-1 α i.n. (E) or PBS i.n. (F) . *P  ≤  0.05, **P  ≤  0.01, ***P  ≤  0.001, ****P  ≤  0.0001. Abundances of *Bacteroidetes* at family level for antibody treated mice and IL-1 α intranasally administered mice (G). Abundances of *Firmicutes* at family level for antibody treated mice and IL-1 α intranasally administered mice (H).

We have previously observed that inappetence following RSV infection alters the gut microbiota and this is reversed by CD8 T cell depletion^18^. Since IL-1α blockade during RSV infection reduced both weight loss and inappetence and IL-1α delivered i.n recapitulates some features of infection, we investigated the impact of IL-1α on the gut microbiome. Feces were collected at days 0 and 7 to sample the gut microbiota. Beta diversity was significantly different following RSV infection (Fig. 6A), indicating a shift in overall gut microbiota composition (*P* < 0.01). There were no differences in beta diversity between days 0 and 7 in αCD8α or aIL-1a treated mice. Beta diversity was significantly different following IL-1α i.n (*P* < 0.001) between day 0 and 1, but no difference was detected in the PBS i.n control mice.

We focused on the two most dominant phyla, *Bacteroidete*s and *Firmicutes*, that we have seen previously change following RSV infection^22^. Consistent with our previous findings^22^, we observed a significant increase in the abundance of *Bacteroidete*s and a significant decrease in *Firmicutes* from day 0 to 7 in the RSV group (Fig. 6B). No significant change in the abundance of *Bacteroidete*s or *Firmicutes* was seen in mice following αCD8α (Fig. 6C) or aIL-1a treatment (Fig. 6D). Feces were collected at days 0 and 1 after administration of recombinant IL-1α. A significant increase in the abundance of *Bacteroidete*s and a significant decrease in *Firmicutes* from day 0 to 1 was seen in the treated group (Fig. 6E). No change in those phyla between days 0 to day 1 was observed in PBS-treated mice (Fig. 6F). At the family level of *Bacterioidetes*, the major change seen after RSV infection or IL-1α instillation was an increase in the abundance of the *Muribaculaceae* family (previously known as the S24_7 family) and *Bacteroidaceae* family (Fig. 6G). For *Firmicutes,* we saw a decrease in the *Lactobacillaceae* and *Lachnospiraceae* families (Fig. 6H). Anti-CD8 or anti-IL-1α treatment reversed the effect of RSV infection on gut bacterial families. Together, these data highlight that IL-1α induces changes in the gut microbiota uncoupled from viral infection.

### IL-1α **is present in the brain following RSV infection and induces weight loss via appetite-associated hormones**

**Fig. 7:**
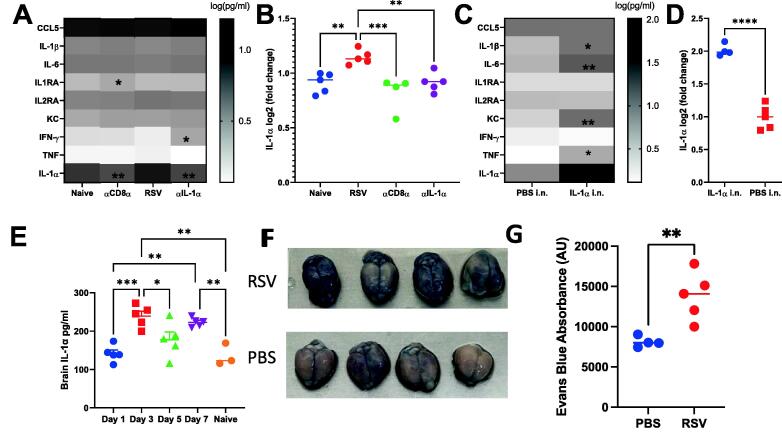
RSV infection alters increases the levels of cytokines in the brain by increasing permeability. Brian supernatants were tested against a panel of cytokines (A, C), with a focus on the IL-1 α level (B, D). For RSV study, brain collected from naïve mice, or α CD8 α , α IL-1 α and or control Ig treated during RSV infection (A, B). For IL-1 α intranasal study, brain collected from PBS i.n. and IL-1 α i.n. treated mice. RNAseq was performed on brains from RSV infected and control (naïve) mice. Brain supernatant was collected on day 1, 3, 5, and 7 following infection and an IL-1 α ELISA was performed (E). 6-8 weeks old mice were infected with RSV and 200μl 2% Evans blue was injected intravenously on day 7 of infection 1 hour before the brains were extracted (F) and Evans blue absorbance was quantified using florescence absorbance excitation 485, emission 680 (G). N≥4, each dot represents an individual mouse (B, D-E, G); or mean +/-SEM (A, C). Significance calculated by ordinary one-way ANOVA and post test. *P  ≤  0.05, **P  ≤  0.01, ***P  ≤  0.001, ****P  ≤  0.0001. Experiment was repeated twice.

As inflammatory cytokines are known to cross the BBB into the CNS to initiate weight loss^31^, we tested whether IL-1α was present in the brain after infection. Brain supernatants from naïve, RSV, αCD8α, or aIL-1a treated mice were tested (Fig. 7A). Cytokines known as inappetence-inducing factors including IL-6, IL-1β, TNF, and IFN-g were not significantly different in the brain after infection, whereas IL-1α was significantly reduced (*P* < 0.0001) in the brain of both αCD8α treated and aIL-1a treated mice compared to RSV treated (Fig. 7B). Measurement of mediators in brain supernatants following IL-1α intranasal administration revealed presence of several cytokines including KC, IL-6, IL-1β, TNF, and IFN-g (Fig. 7C). These cytokines were increased compared to PBS intranasal control with notably IL-1α 2-fold upregulated (Fig. 7D). Having seen an influx of IL-1α into the brain, we investigated the kinetics of IL-1α in the brain during RSV infection. Interestingly, two peaks of IL-1α level were identified at days 3 and 7 (Fig. 7E). A previous study has observed that viral infection increases BBB leakiness after infection, which may lead to the influx of cytokines seen^32^. To test whether a similar effect was seen at the peak of infection-caused weight loss (day 7), we measured the level of Evans blue dye in the brain 30 minutes after an IV injection. RSV-infected mice visually had more dye in the brains (Fig. 7F), which gave a significantly greater absorbance when quantified by fluorescence assay on brain supernatants (Fig. 7G).

**Fig. 8:**
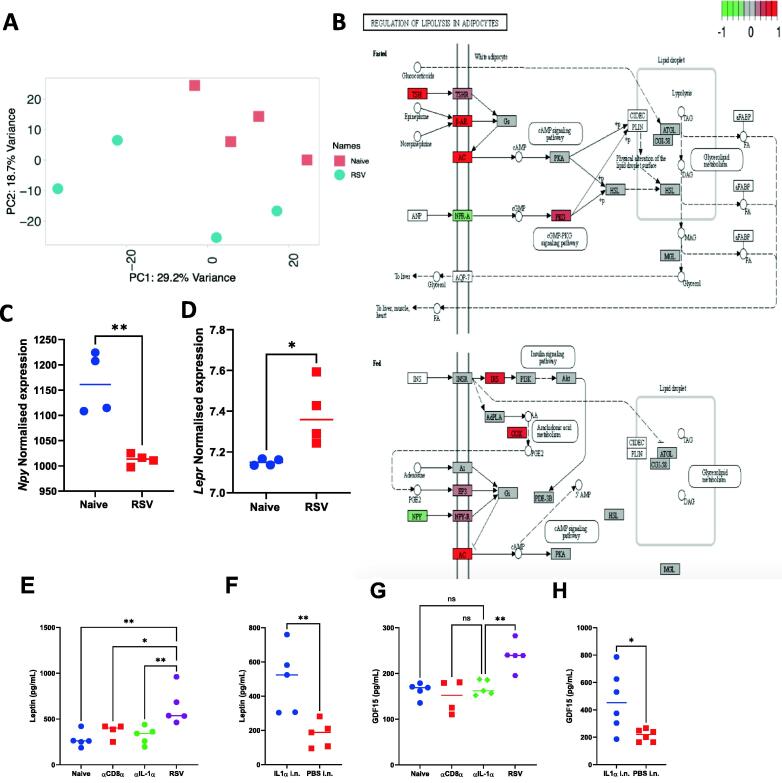
Brain transcriptomics revealed a role of leptin and GDF15 in weight loss following RSV infection. Principal component analysis was carried out to identify batch effects (A) and KEGG pathway analysis of differentially expressed genes termed Regulation of lipolysis in adipocytes (B). Normalised expression values taken from the Day 7 post RSV comparison for RNA levels of *Npy* (C) and *Lepr* (D) . Leptin (E, F) and GDF15 (G. H) measured in serum by ELISA from Naïve, α CD8 α , α IL-1 α and RSV (E, G) and PBS i.n. or IL-1 α i.n. treated mice (F, H). N≥5, each dot represents an individual mouse (C-H). Significance calculated by ordinary one-way ANOVA and post test. *P  ≤  0.05, **P  ≤  0.01, ***P  ≤  0.001, ****P  ≤  0.0001.

To identify whether there were any changes in the brain transcriptome after RSV infection that might indicate how infection leads to inappetence, we performed RNAseq on the brain of mice at day 7 post-infection. The transcriptomic signature formed distinct clusters between RSV-infected brain and naïve brain (Fig. 8A). There were 68 DEG between RSV and naïve (Supplementary Table 9); they were associated with an upregulation of T cell and NK mediated genes (*H2-Q4*, *H2-K1*, *H2-D1*, *H2-T23*, *Iigp1*), Guanylate binding proteins (*Gbp2*, *Gbp2b*, *Gbp3*, *Gbp4, Gbp5, Gbp7*), Neutrophil associated genes (*Ly6a*), TNF pathway (*Tnfsf10*) and Antiviral response (*Ifitm3*, *Oasl2*). Performing Kyoto Encyclopedia of Genes and Genomes (KEGG) pathway analysis on the 38 DEGs revealed that RSV infection induced significant changes in the pathways “regulation of lipolysis in adipocytes”. Thyroid-stimulating hormone (*Tsh*), which has been shown to reduce food intake when injected cranially into rats^33^, was significantly upregulated in the RSV-infected mice (Fig. 8B). Transcript levels of neuropeptide Y (*Npy*), a hormone that induces appetite, were reduced upon RSV infection (Fig. 8C)^34^, whereas leptin receptor (*Lepr*) transcripts were increased upon RSV infection (Fig. 8D), both of these genes are implicated in reducing food intake^35^, ^36^. Reflecting the brain RNA upregulation, in the RSV group we observed a significant increase in leptin protein levels in the blood compared to aIL-1a or αCD8α (Fig. 8E). In the IL-1α i.n administered group, a significant increase in leptin was seen compared to PBS (Fig. 8F). GDF-15, GDF-15 a cytokine associated with weight loss and poor outcomes in patients critically ill with COVID-19^37^, ^38^, was also higher in serum from the RSV-infected group compared to αCD8α or aIL-1a treated mice (Fig. 8G). GDF-15 was higher in the IL-1α intranasal group compared to PBS (Fig. 8H).

## DISCUSSION

The main goal of this study was to dissect the immune mechanism of RSV infection-induced weight loss. Similar to previous observations, depletion of CD8^+^ T cells reversed weight loss but increased viral load^18^. Multi-organ system analysis at the peak of RSV infection-induced weight loss showed significant changes in the blood, lung, and brain transcriptomes. Gene expression profiles were largely organ-specific, but common mechanisms were also observed. In the absence of CD8 T cells, there was an upregulation in metabolism-related genes for example *Pcx* and *Scd1*, important for central insulin and glucose homeostasis in adipose tissue. It is therefore tempting to speculate that CD8^+^ T cell-induced weight loss may represent a conserved response to tissue damage or excessive inflammation upon infection.

Using a combination of transcriptional profiling and experimental validation, we report a role for IL-1α in weight loss following RSV infection. We did not see a role for IL-1β, a cytokine often associated with mediating weight loss. This suggests that IL-1α and IL-1β have different functions despite binding to the same receptors and inducing similar cellular responses. A functional difference between IL-1α and IL-1β has been reported for several infections, including group A *Streptococcus*^39^ and *Toxoplasma gondii*^40^. In our RSV model, IL-1β was required for disease resolution as IL-1β blockade exacerbated weight loss. We have previously observed that RSV SH blocks IL-1β^26^. However, we show that IL-1α neutralization reduced RSV infection-induced weight loss. In our RSV model, the peak of weight loss was associated with the peak CD8^+^ cell recruitment to the lung. When we delayed CD8 recruitment using FTY720 we saw an equivalent delay in weight loss. We suspect during RSV infection that cleavage of pro-IL-1α from the infected cells is achieved through granzyme B (GzmB), a key protease of CD8^+^ T cells^41^. More work however is required to determine this interaction.

We propose IL-1α as a significant mediator of systemic inflammation ultimately leading to a weight loss response. We saw a CD8^+^-dependent response consistently in the blood, lung, and brain signature following RSV infection. While PGSEA analysis highlighted the IL-1 pathway in the blood transcriptome comparing RSV control to aCD8a group, a similar pathway was not observed directly in the lung. This might reflect the complex nature of the interplay between the host immune response to RSV locally and systemically. As IL-1a is constitutively produced in mature and immature forms, it has a pleiotropic role as an alarmin and as a pro-inflammatory cytokine. Thus, the extent of CD8^+^ T cell depletion in lung injury reflected at the mRNA level might not be as obvious.

Key metabolism pathways emerged from the lung transcriptome of IL-1α depleted mice following RSV infection, in particular sphingolipid signaling, glycolysis, fatty acid synthesis, and adipocytokine signaling pathways. All these pathways have been shown to be important for tissue wasting and weight loss. Intranasally administering IL-1α in the absence of a live pathogen also led to weight loss, accompanied by a transcriptomic signature in the lung with overwhelming similarity to RSV infection. This suggests that IL-1α plays an indispensable role during RSV infection-induced weight loss. As pro-inflammatory cytokines are well-documented to be involved in dysregulation of muscle and fat metabolism in pathophysiology of chronic inflammatory disease and cancer, we also explored the cytokine profile in the brain. Significantly more IL-1α was present in the brain after RSV infection. A similar increase was seen after IL-1α intranasal delivery, though the intranasal delivery may have crossed into the brain directly through the olfactory mucosa rather than from the lungs and circulation. We hypothesize that IL-1α in the brain regulates inappetence through the IL-1R1 receptor found in the CNS activating appetite-associated hormones including neuropeptide Y and leptin.

Why does the immune response to infection lead to a decrease in appetite and subsequent weight loss? It has been suggested that weight loss has evolved as a strategy to limit nutrient availability to the virus and counteract viral replication without having a direct adverse effect on pathogen clearance^10^, ^42^. Weight loss, however, carries an evolutionary trade-off since long-term weight loss can become pathological and eventually compromise host homeostasis. Another hypothesis is that changes in dietary patterns affect the gut microbiome which then shapes the immune response to infection^18^. The pattern of gut microbiota change observed for RSV infection was also seen following IL-1α intranasal administration and reversed by IL-1α blockade suggesting a conserved change in the gut microbiome when food intake was reduced.

Preventive options for infants and the elderly against RSV continue to improve with a new monoclonal antibody and vaccines being licensed^43^. But these will not necessarily be available to all, especially in low-income countries where the burden of disease and death is greatest, alternative low-cost interventions are important. Severe weight loss is often seen in infants and elderly infected with RSV. Weight loss has been identified as a negative outcome of respiratory viral infection, leading to reduced lung function, lowered tolerance to mechanistic and invasive care, and decreased survival rates. Therefore, treating weight loss could be a potential treatment option. By revealing IL-1α-dependent weight loss following RSV infection, our data suggests a potential way that IL-1α changes host behavior. This demonstrates that the host response to respiratory viral infection, rather than the direct effects of virus replication, dictates the type and extent of injury incurred^44^.

Although the translational importance of IL-1α in our RSV model is yet unclear, the IL-1α monoclonal antibody, MABp1, has shown prominent clinical benefits in a trial treating cancer-associated cachexia^45^. Another monoclonal neutralizing antibody targeting IL-1α, Bermekimab, was used in a randomized, placebo-controlled study to evaluate the efficacy of the treatment in 333 patients with advanced, metastatic colorectal cancer^46^. Following an 8-week monotherapy course, increased lean body mass, decreased constitutional symptoms, and improved quality of life were observed in treated patients. Monoclonal antibodies targeting other anorexia and inappetence-inducing factors identified in this study have also been investigated for disease-associated cachexia, such as GDF-15^47^ and leptin^48^ in mice models. Blocking inducers of inappetence with monoclonal antibody treatment could therefore mitigate the symptoms of infection that arise independently of pathogen burden. Not knowing when a patient was infected has been a challenge with immune interventions which can run the risk of increasing viral load if made in earlier stages of the infection when pathogen control is required. Our demonstration that there is a separation of weight loss and viral load makes targeting the appetite hormones during infection an attractive option because it could be used agnostically of onset date of infection.

## METHODS

### Animals

The 6–8 weeks old female BALB/c specific-pathogen-free (SPF) mice were purchased from Charles River Laboratories (Portscatho, U.K.) and maintained according to institutional and Home Office guidelines. All experiments were performed in the SPF room in the animal facility on a 12-hour light/dark cycle at 20°C to 24°C with 55% ± 10% humidity at Imperial College London, St Mary’s Hospital Campus. All work was approved by the Animal Welfare and Ethical Review Board at Imperial College London, and studies were in accordance with the Animal Research: Reporting of In vivo Experiments (ARRIVE) guidelines.

Mice were housed in groups of five animals per cage the exception being for food intake studies when mice were kept in groups of two. For RSV infection studies, mice were anesthetized using 2.5%–3% of isoflurane and i.n infected with 3.7 × 10^5^ or 1 × 10^6^ plaque-forming units (PFU) RSV subgroup A2 as indicated in the figure legend. For IL-1α recombinant protein experiment, mice were i.n introduced to 3 mg of mature IL-1α in 20 μl PBS (Sino Biological InC, Texas, USA, LC10OC3102). Food intake and water intake were measured during specific experiments by weighing the contents of the food or water bottle respectively at the same time every day. Each food weight was then divided accordingly by the number of mice in each cage.

### RSV culture

Stocks of RSV A2 were stored in liquid nitrogen and thawed on the day of inoculation. Virus was propagated on HEp2 cells with serum-free Roswell Park Memorial Institute **(**RPMI) media. The virus-containing flask was left at 37°C for 2 hours and rotated every 15 minutes to counteract any uneven surface in the incubator. At 2 hours, the flask was then supplemented with 10% of Fetal Bovine Sera (FBS) and left in the incubator for 24 hours. On day 2, FBS was reduced to 2% and left for a further 24 hours. Cytopathic effect was assessed visually and when 50% was achieved, virus stocks were pooled together and frozen at -80°C until further use.

### Monoclonal antibody depletion and FTY720 treatment

Mice were intraperitoneally (i.p) injected with 500 μg monoclonal anti-mouse CD8α antibody (Clone:53-6.7, BioXCell), IL-1α (Clone: ALF-161, BioXCell, Lebanon, USA), IL-1β (Clone:B122, BioXCell), or HRPN isotype control (HRPN, BioXCell) in 500µl. For the CD8α depletion, mice were i.p. injected on days −1, 2 and 5 relative to infection. In comparison, cytokines were injected at days −1, 1, 3, and 5, relative to infection.

For FTY720 treatment, FTY720 (6176, BioTechne,Minneapolis, MN, USA) was diluted in PBS to a final concentration of 25μg in 250 μl PBS. The FTY720 was administered i.p 24 hours before RSV infection and then every 24 hours thereafter^49^.

### BAL

After cutting open the abdominal cavity, the diaphragm was carefully punctured. The skin and tissue were removed from the throat until the trachea was revealed. A cut was inserted into the trachea using a sharp blade and an 18-gauge polypropylene gavage tube (with the tip removed: FTP-18–30: , Instech, Leicestershire, UK) inserted into the incision. The mouse lungs were ravaged to inflate the lung three times with 1 ml of PBS and transferred to a 1.5 ml Eppendorf tube. The BAL fluid was centrifuged at 1500 rpm for 5 minutes and supernatants were collected and frozen at -80°C. The cell pellet was resuspended with 500 µl of Ammonium–Chloride–Potassium (ACK) lysis buffer for 2 minutes then quenched with PBS prior to centrifugation at 1500 rpm for 5 minutes prior to resuspending in RPMI for flow cytometry.

### Tissue dissection and cell processing

Mice were culled by administration of 100 μl intraperitoneal pentobarbitone (20 mg dose, Pentoject, Animalcare Ltd.,York, UK) and blood was collected from femoral veins. For RNAseq processing, 1 ml blood was immediately mixed with 2.76 ml of PAXgene collection reagent (PreAnalytiX GmbH, Switzerland) and stored at −80°C prior to subsequent RNA extraction. For enzyme-linked immunosorbent assay (ELISA) analysis, sera were isolated from blood after clotting by centrifugation and frozen for future analysis.

Lungs were removed after dissection with the right lobe processed for flow cytometry and the left lobe frozen for future RNA extraction. For flow cytometry, tissue was homogenized by passage through 100 mm cell strainer followed by centrifugation at 200 x g for 5 minutes. Supernatants were removed and treated with red blood cell lysis (ACK) buffer (0.15 M ammonium chloride, 1 M potassium hydrogen carbonate, 0.01 mM EDTA, pH 7.2). Reaction was then quenched with PBS and centrifuged at 200 × g for 5 minutes. The cell pellet was resuspended in RPMI 1640 medium with 10% FCS, and viable cell numbers were determined by trypan blue exclusion.

Brains were collected from mice after dissection and each brain was homogenized by passage through a 100 mm cell strainer followed by centrifugation at 200 x g for 5 minutes. Supernatants were collected and stored at −80°C until required. In a separate study, mouse brains were snap-frozen and processed for RNA.

### Viral plaque assay using Vero cells

For this, 1 × 10^5^ Vero cells were seeded per well of 24 well plates the night before infection in 37°C 5% CO_2_ incubator until 90% confluent on the day of infection. Then, 100 μl of lung mixture (after passage through 100 mm cell strainer directly) and a 5-fold dilution were plated in triplicate wells and incubated in the incubator for 1.5 hours (shaking every 15 minutes). After the inoculum was aspirated, 1 ml of overlay (1.2% Avicel (RC-591NF, UPI Inc., Mebane, NC, USA) in distilled water and 1:1 2 × DMEM) was added per well for 3 days before staining. On the day of staining, the overlay was removed and 1 ml of freezer-cold fixing solution (0.6% hydrogen peroxide in methanol) was added for 30 minutes. The plates were washed with PBS before adding 1 ml of 5% (w/v) milk in PBS blocking for 1 hour on rocker at room temperature. The blocking solution was removed and replaced with 200 μl of 1/2000 dilution of RSV monoclonal antibody (Chemicon Int, MAB858-4, Texas, USA) followed by secondary antibody (HRP-conjugated Goat anti-mouse IgG, 074-1806, Merck KGaA, Darmstadt, Germany) incubated for 1 hour each on a gentle rocker at room temperature. Plates were washed with PBS and then TrueBlue Peroxidase substrate (5510-0030, KPL Seracare, Milford, MA, USA) with 30% Hydrogen peroxide for staining and counting.

### Blood-brain permeability by Evans blue

Seven days after RSV infection, mice received 200 μl of 2% Evans blue IV. Brains were collected and mashed through a 70 μm filter in 1ml PBS. Absorbance was measured at 485 excitation and 680 emission in a FLUOstar Omega reader and plotted as Absorbance Units (AU) blanked with (no Evans blue) brain supernatant.

### Flow cytometry

Live lung cells and cells from BAL were plated out onto a U-shaped 96-well plate and then spun down at 2000 rpm for 2 minutes at 4°C. Then, 100 µl of Live/Dead violet dye (L34961, Invitrogen^TM^, Carlsbad, California, USA) was added for 20 minutes at 4°C in the dark, the plate was then centrifuged at 2000 rpm for 2 minutes and the supernatant taken off. The cell pellet was resuspended in Fc block (Clone: 2.4G2, BD Biosciences, Berkshire, UK) in PBS-1% bovine serum albumin (BSA) and stained with the following surface antibodies (all BD) CD3 (Clone: 17A2, FITC), CD8 (Clone: 53-6.7, APC-H7,), CD4 (Clone: rm4-5, PE-Cy5.5, RSV Pentamer (SYIGSINNI, PE: ProImmune, Oxford, UK) for 1 hour in the dark. Excess antibodies were washed off with 1% BSA in PBS three times before being filtered through the FACS tubes on an LSR Fortessa Flow cytometer (BD Biosciences, Piscataway, NJ, USA) and analysed with software from FlowJo LLC (Oregon, USA). Fluorescent minus one controls were used for surface stains. Analysis was performed using FlowJo and gated as shown in Supplementary Fig. 1.

### qPCR

RNA was extracted from the left lung lobe by first homogenizing the tissue using a TissueLyzer (Qiagen, Manchester, UK) at 50 oscillations for 4 minutes followed by a TRIzol (QIAzol, 79306; Qiagen) and chloroform extraction. RNA concentrations were determined using a Nanodrop before converting into cDNA using a GoScript reverse transcription system (Product code A5001; Promega, Southampton, UK) and 2µg for all samples. qPCR for the RSV L gene was performed on a Stratagene Mx 3005p (Agilent Technologies, Santa Clara, CA, USA) using the primers 5′-GAACTCAGTGTAGGTAGAATGTTTGCA-3′ and 5′-TTCAGCTATCATTTTCTCTGCCAA-3′ and probe 5′-6-carboxyfluorescein (FAM)-TTTGAACCTGTCTGAACAT-6-carboxytetramethylrhodamine (TAMRA)-3′^50^. RNA copy number per mg of lung RNA was determined using an RSV L gene standard. Gene expression levels of the RSV L gene were normalized to the Glyceraldehyde 3-phosphate dehydrogenase (GAPDH) copy number.

### Cytokine detection by ELISA or multiplex

Microtiter plates were coated overnight with 100 μl of a 1:500 dilution of either RSV, mouse IL-1α (MOFI00057, AssayGenie, Dublin, Ireland), mouse leptin (MOB00B, biotechne), mouse GDF15 (MOFI00042, AssayGenie) or mouse Kappa and lambda antigen (standards). The plate was then washed three times with PBS + 0.05% tween before being blocked with 1% BSA for 1 hour at 37°C. Dilutions of test samples were added for a further 2 hours. Bound antibody was detected using peroxidase-conjugated rabbit anti-mouse IgG (1:5000 dilution). Color was developed using TMB substrate for 3 minutes and stopped with H_2_SO_4_. Plates were read on the FLUOstar Omega machine (BMG LABTECH, Aylesbury Bucks, UK).

A multiplex cytokine panel, which included IL-1α, IL-1β, CCL5, IL-6, IL1RA, IL2RA, KC, IFN-g, TNF-α, was designed with AssayGenie (Dublin, Ireland). Briefly, 45 μl of capture bead working suspension was added to each well on a 96-well plate for an hour and buffers in the wells were removed using a filter plate washer connected to a vacuum source. Then, 30 μl of assay buffer was added to each well and left to coat for an hour. After that, 15 μl of brain supernatant or standards were added to each well and the plate sealed and incubated with shaking for an hour at room temperature. The filter plate washer was used to empty solution from the wells which were then washed with wash buffer three times before adding 25 μl of biotinylated antibody working solution to each well. The plate was incubated for 30 minutes at RT and then washed three times after which 25 μl of streptavidin-PE working solution was added to each well with incubation at RT and shaking for 20 minutes. After three further wash buffer washes, 300 μl of reading buffer was added to each well which was then read on a flow cytometer. Downstream analysis was performed by AssayGenie.

### RNA extraction, processing, and normalization

Blood was collected and mixed with PAXgene solution, using the PAXgene blood RNA kit according to the manufacturer’s instructions (Qiagen). Lung RNA and brain RNA were extracted using QIAzol (Qiagen) and chloroform extraction. RNA QC and library preparation were performed by Novogene using the Illumina HiSeq at a target depth of 50 million 100–150 bp pair-end reads per sample. Quality of Raw RNAseq reads generated was assessed using FastQC (v0.11.9) to ensure good quality scores, GC content and no adaptor reads, then appropriate adjustment was made using the program Trimmomatic (v1.0.40). Raw reads were then mapped to Mouse Reference Genome (Gmc38) using STAR (Spliced Transcripts Alignment to a Reference, v6.2.0), and count data for each gene was performed using Salmon (v1.2.0). PCA and heatmap visualization on normalized sequence data were analyzed by the variance stabilizing transformation method. R function prcomp() was used for PCA in the package devtools (v2.4.2) and heatmap visualization was performed using the heatmap.2 function in gplot package (v3.1.1).

### Differential expression analysis

Differential gene expression analysis was performed either using DeSeq2^51^ differential analysis to obtain a list of genes, *p* values, adjusted *p* values, or log2 fold changes with positive log fold change values indicating increased gene expression negative values decreased gene expressed. False discovery rate was calculated by applying the weighted Benjamin–Hochberg method for multiple hypothesis testing. A gene was considered differentially expressed if the absolute fold change was above 0.5 with adjusted *p* value <0.05.

### Gene ontology and KEGG network analysis

ClusterProfiler^52^ was used to assess the enrichment of Gene Ontology (GO) pathways in each gene list. Network analysis of the GO terms was analyzed using the emapplot function in the clusterProfiler package. KEGG pathway was analyzed using the gseKEGG function. Gene lists analyzed include genes that were identified as significantly differentially variable and genes that were in a specific GO term. GSEA was performed where enrichment score was calculated as the −log10 (*p* value)^53^. The network analysis of KEGG pathways (network visualization and clustering) was performed with NetworkAnalyst^54^. R code is available upon request.

### Microbiome

Mice fecal samples were collected under sterile conditions on day 0 and day 7 after RSV infection for depletion studies, or day 0 and day 1 for IL-1α i.n studies. Briefly, bacterial DNA was extracted from 30 mg feces/mouse/time point using the FastDNA spin kit for soil (116560200; MP Biomedicals, Loughborough, UK). Control extraction with no sample was performed for each extraction batch and sequenced to monitor any bacterial DNA contamination during the extraction and within the kit components. In addition, each sequencing run included a negative control (nuclease-free water), a positive control (mock community), and a FastDNA spin kit control. The V4 variable region of the 16S rRNA was amplified using universal bacterial primers, each uniquely barcoded per sample (Illumina Nextera Indexes version 2). The amplicons were purified, quantified, and equimolar pooled to produce a 16S rRNA gene library as described previously^22^. The 8 pM denatured library was spiked with 20% PhiX and paired-end sequencing performed using the Illumina MiSeq platform. After that, 16S rRNA gene sequencing data were processed using the QIIME2 software (2021.11). Taxonomy was assigned and clustered using the SILVA database and Amplicon Sequence Variant ASVs were assigned using the Scikit Learn packages in the QIIME2 environment. Diversity and phylogenetic analysis were conducted in RStudio using phyloseq and vegan packages. Beta diversity was analyzed using the nonmetric multidimensional scaling (NMDS) ordination on a Bray-Curtis dissimilarity matrix.

### Statistics

All statistical analyses were performed in Graph Pad Prism V9 (GraphPad Software, San Diego, CA), R version 3.5.0. A statistically significant difference was defined as a *p* value <0.05 by one-way analysis of variance. Data has been archived at ArrayExpress (RNAseq) and European Nucleotide Archive (Microbiome).

## AUTHOR CONTRIBUTIONS

ZW: Data Curation, Formal Analysis, Visualization, Investigation, Writing – original draft; LC: Investigation; HJS: Investigation, CT: Investigation, LGM: Investigation, HL: Investigation, KK: Investigation; MM: Supervision, Writing – editing; JST: Conceptualization, Funding acquisition, Writing – review & editing.

## DECLARATIONS OF COMPETING INTEREST

The authors have no competing interests to declare.

## FUNDING

This work was funded in part by Department of Infectious Disease, Imperial College London as part of the Molecular and Cellular Basis of Infection program. The work was also supported by the Wellcome Trust studentship 102126/B/13/Z.

## Appendix A Supplementary data

Supplementary Figs. 1-3

Supplementary Table 1 Respiratory syncytial virus versus Naïve differentially expressed gene (blood).

Supplementary Table 2 Respiratory syncytial virus versus anti-cluster of differentiation 8 differentially expressed gene (blood).

Supplementary Table 3 Common differentially expressed gene respiratory syncytial virus and anti-cluster of differentiation 8 (blood).

Supplementary Table 4 Respiratory syncytial virus versus naïve differentially expressed gene (lung).

Supplementary Table 5 Respiratory syncytial virus versus anti-CD8 differentially expressed gene (lung).

Supplementary Table 6 Respiratory syncytial virus versus anti-IL1a differentially expressed gene (lung).

Supplementary Table 7 Common differentially expressed gene (lung).

Supplementary Table 8 Overlap interleukin-1 IN and respiratory syncytial virus (lung).

Supplementary Table 9 Respiratory syncytial virus versus naïve differentially expressed gene (brain).
