# Supplementary material for: IL-1α is required for T cell-driven weight loss after respiratory viral infection
Source: Mucosal Immunol. 2024 Apr;17(2):272–87. doi: 10.1016/j.mucimm.2024.02.005 (PMC11009121; doi:10.1016/j.mucimm.2024.02.005)
Supplement: Supplementary Figs. 1-3 [file mmc1.pptx]

## Slide 1
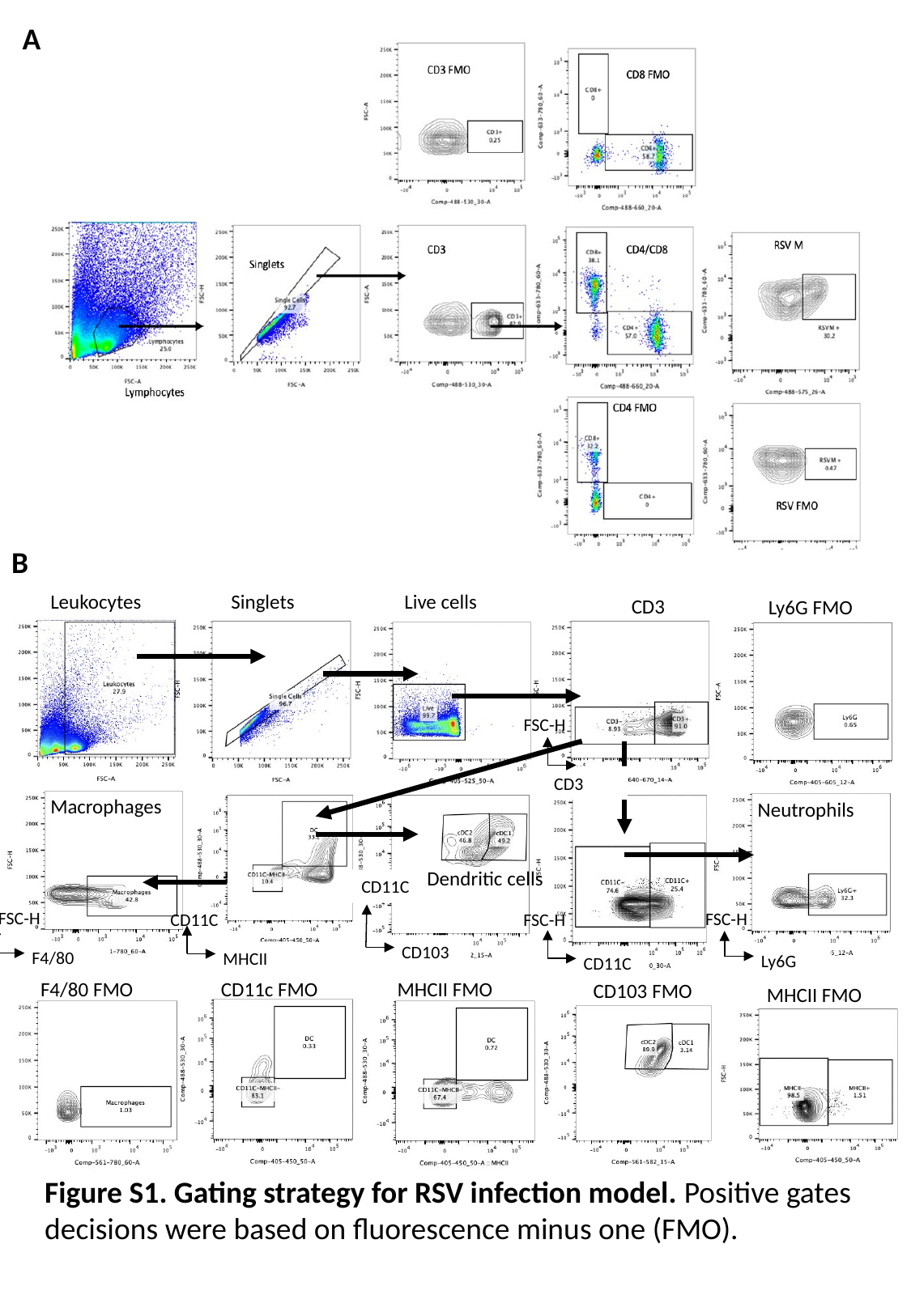

A
B
Singlets
Live cells
Leukocytes
CD3
Ly6G FMO
FSC-H
CD3
CD11C
FSC-H
FSC-H
CD11C
FSC-H
CD103
F4/80
MHCII
Ly6G
CD11C
F4/80 FMO
CD11c FMO
MHCII FMO
MHCII FMO
Macrophages
Neutrophils
Dendritic cells
CD103 FMO
Figure S1. Gating strategy for RSV infection model. Positive gates decisions were based on fluorescence minus one (FMO).

## Slide 2
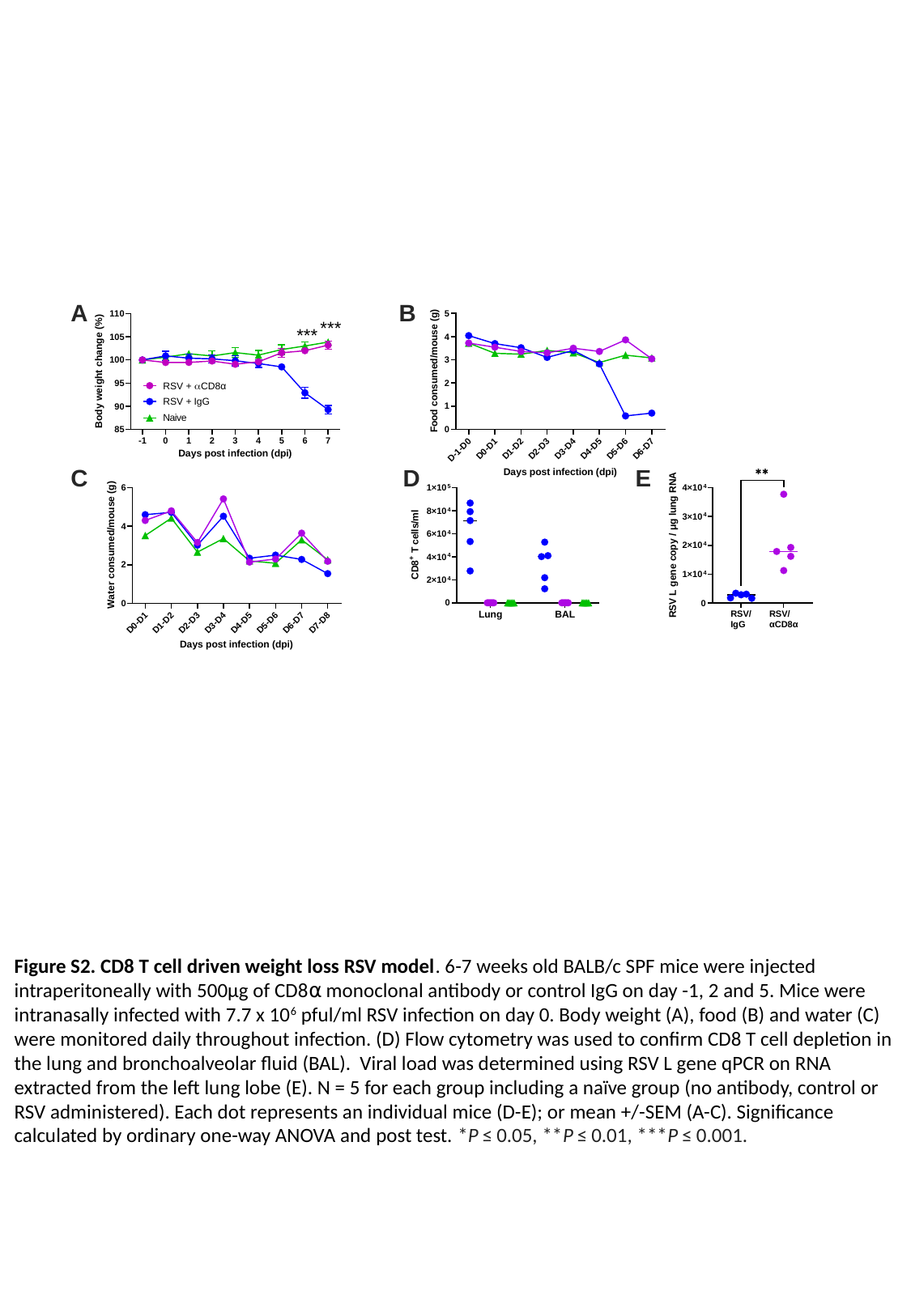

Figure S2. CD8 T cell driven weight loss RSV model. 6-7 weeks old BALB/c SPF mice were injected intraperitoneally with 500µg of CD8⍺ monoclonal antibody or control IgG on day -1, 2 and 5. Mice were intranasally infected with 7.7 x 10^6^ pful/ml RSV infection on day 0. Body weight (A), food (B) and water (C) were monitored daily throughout infection. (D) Flow cytometry was used to confirm CD8 T cell depletion in the lung and bronchoalveolar fluid (BAL). Viral load was determined using RSV L gene qPCR on RNA extracted from the left lung lobe (E). N = 5 for each group including a naïve group (no antibody, control or RSV administered). Each dot represents an individual mice (D-E); or mean +/-SEM (A-C). Significance calculated by ordinary one-way ANOVA and post test. *P ≤ 0.05, **P ≤ 0.01, ***P ≤ 0.001.

## Slide 3
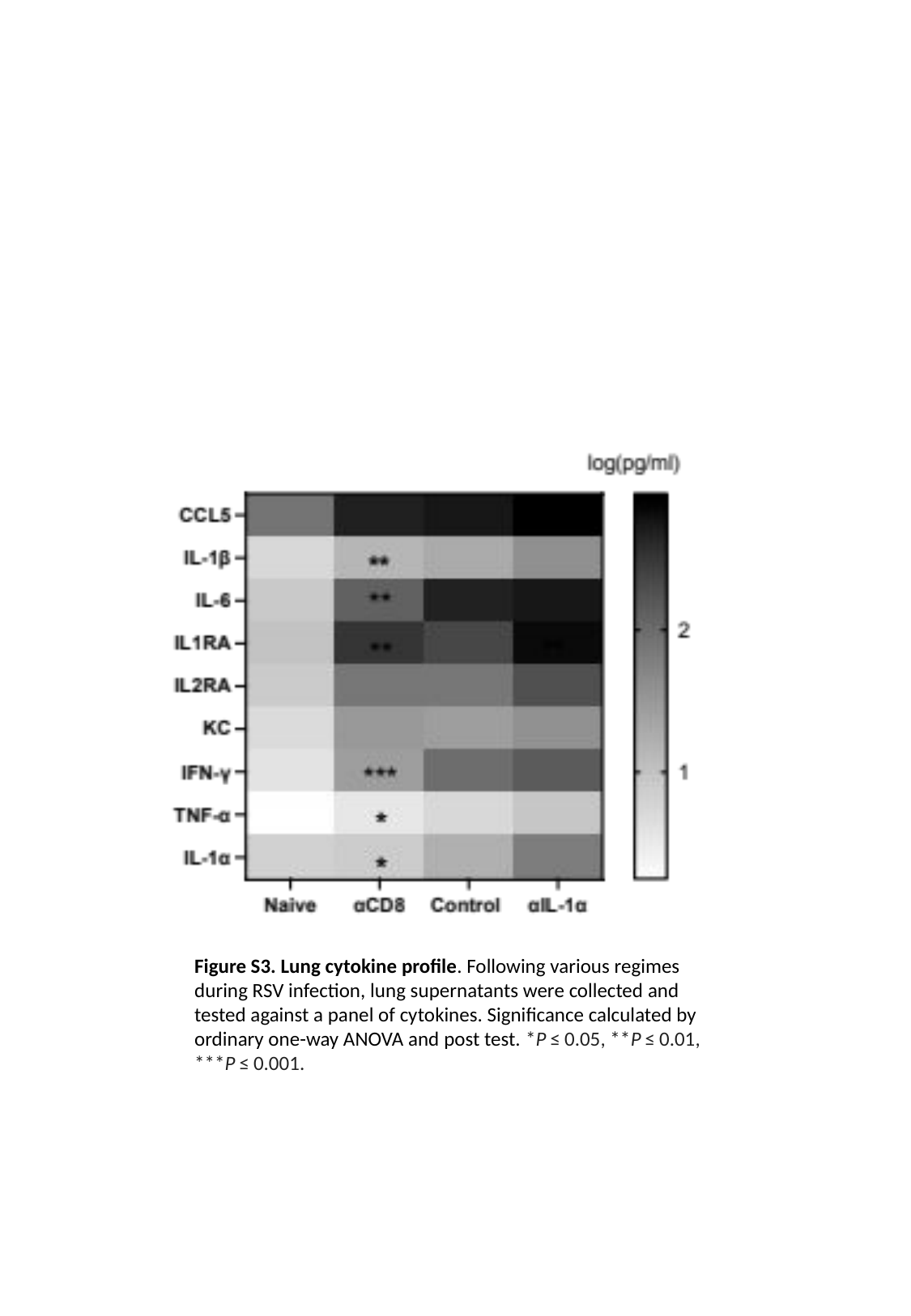

Figure S3. Lung cytokine profile. Following various regimes during RSV infection, lung supernatants were collected and tested against a panel of cytokines. Significance calculated by ordinary one-way ANOVA and post test. *P ≤ 0.05, **P ≤ 0.01, ***P ≤ 0.001.
